# *Aspergillus awamori* MK788209 cellulase: production, statistical optimization, pea peels saccharification and textile applications

**DOI:** 10.1186/s12934-023-02286-w

**Published:** 2024-01-05

**Authors:** Faten A. Mostafa, Hala R. Wehaidy, Samar Sharaf, Heba M. El-Hennawi, Safia A. Mahmoud, Shireen A.A. Saleh

**Affiliations:** 1 Chemistry of Natural and Microbial Products Department, National Research Centre, Dokki, Giza, 12622 Egypt; 2 Preatreatment and finishing of cellulosic based fabric Department, National Research Centre, Dokki, Giza, 12622 Egypt; 3 Dying, Printing and Textile Auxiliaries Department, National Research Centre, Dokki, Giza, 12622 Egypt

**Keywords:** Lignocellulosic wastes, Solid state fermentation, Fungal cellulase, Saccharification, Biopolishing, Desizing

## Abstract

**Background:**

The demand for low-cost cellulolytic enzyme synthesis is rising in the enzyme market. This work aims to produce cellulase by utilizing various agricultural wastes and investigating the use of enzyme in saccharification and textile industries.

**Results:**

Solid state fermentation (SSF) was applied to produce industrial enzymes, particularly cellulase, through utilizing Molokhia (*Corchorus olitorius*) stems by *Aspergillus awamori* MK788209 isolate. Two stages of statistical factorial designs Plackett-Burman (PB) and Central Composite Design (CCD) were applied to enhance the *A. awamori* MK788209 cellulase production from Molokhia stems (MS). The fold increase of enzyme production by PB followed by CCD was 2.51 and 4.86, respectively. Additionally, the *A. awamori* MK788209 culture filtrate was highly effective in saccharifying various agricultural wastes, particularly pea peels (PP) (yielding 98.33 mg reducing sugar/ml), due to its richness in cellulase, laccase, xylanase, pectinase, and amylase. By optimizing the three main variables; pea peel weight, culture filtrate volume added, and saccharification time by CCD, the sugar recovery from PP was enhanced, leading to a 3.44-fold increase in reducing sugar recovery (338 mg reducing sugar /ml). Furthermore, the *A. awamori* MK788209 culture filtrate showed high efficacy in textile applications, enhancing the roughness, weight loss, white index, and printing capability of treated cotton fabrics.

**Conclusions:**

*A. Awamori* MK788209 produced cellulase which was effective in PP saccharification. The enzyme was also capable of enhancing cotton fabric properties.

## Background

The production of valuable products using inexpensive, environmentally friendly resources is today’s main target. Because of this, there is a lot of attention paid to the use of lignocellulosic agricultural wastes, which are primarily responsible for environmental contamination because of their accumulation and high disposal costs. Lignocelluloses are mainly composed of cellulose (35–50%) (homopolysaccharide of β-1, 4-glucan), hemicelluloses (25–30%) (heteropolysaccharides of xylans, mannans) and lignin (25–30%) (complex polyphenolic structure). Currently, pectinase, xylanase, and cellulases account for over 20% of the global enzyme market. High production cost and low production yields caused the bottleneck for industrial enzymes applications [1, 2].

Thus, these lignocellulolytic wastes can be used as fermentation substrates for microbes [3, 4] for the production of lignocellulolytic enzymes that are utilized in, textile industry [5, 6], paper, fruit and vegetable processing industries [7], and agricultural inputs [8]. Alternatively, these wastes can be transformed into valuable products by employing enzymes like cellulases, hemicellulases, glucanases, polysaccharide lyases, glycosidase hydrolases, and carbohydrate esterases [9]. Fuel and chemicals are two of these valuable products [10]. Some authors [11, 12] have described the biological conversion of agricultural wastes into organic materials, the use of microorganisms to bioremediate environmental pollutants and interact with the root rhizosphere to promote plant growth and soil structure, resulting in high-value and low-cost bioorganic farm inputs [13–15].

Nevertheless, three cellulolytic enzymes, endoglucanases (EC 3.2.1.4), exoglucanases (cellobiohydrolases; EC 3.2.1.91), and β-glucosidases (EC 3.2.1.21), must work in harmony to bioconvert cellulose to fermentable sugars by releasing glucose from cellobiose [16–19]. Numerous microorganisms, such as bacteria, actinomycetes, and fungi, produce cellulases; however, because fungal cellulases are excreted extracellularly, they are of particular interest [20].

Converting lignocellulolytic wastes into ethanol primarily involves two steps: first, the cellulose in the lignocellulosic biomass is hydrolyzed to form reducing sugars, and then the sugars are fermented to produce ethanol. Since enzyme hydrolysis is often carried out in mild conditions (pH 4.8 and 45–50 °C) and does not have a corrosion problem, it is less expensive than acid or alkaline hydrolysis [21].

Moreover, lignocellulolytic enzymes are implicated in textile industry. For example, desizing (removing starch size with amylases); bioscouring (using pectinase, lipase, and cellulase solutions to dissolve waxes, proteins, pectins, and natural fats from the surface of cotton fibers); bio-finishing (using cellulases to remove fiber fuzz and pills from the substrate surface to improve the appearance of cotton fabrics and garments); biostoning (using cellulases to stonewash denim fabrics to create the trendy aged appearance); laundering garments or removing different types of stain with a mixture of enzymes [22].

The goal of this study was to use cheap, widely accessible lignocellulosic waste for solid state fermentation by a fungal isolate, which would produce cellulase. Improvement of cellulase yield through two stages of statistical design (Placket-Burman design and Central composite design). Saccharification of eight agricultural wastes using crude lignocellulolytic enzymes. In order to maximize the yield of reducing sugars, it was also investigated how different factors, including pea peel weight, enzyme load, and saccharification period, affected the enzymatic hydrolysis of pea peel into reducing sugar. Additionally, the usefulness of fungal cellulase at various stages of the textile industry was studied.

## Materials and methods

### Raw material

Agro-residues (corn cobs: CC, lemon peels: LP, potato shells: PS, okra suppression: OS, molokhia stem: MS, rice straw: RS, onion peel: OP and pea peels: PP) were collected from local market in Egypt. They were washed and dried in an oven (70 °C for 24 h). To be used as the substrate in SSF, the dried materials were ground using an electric grinder, separated using a 1 cm sieve, and then sealed in airtight containers. Sigma-Aldrich provided the soluble starch, xylan, pectin, ABTS, and carboxymethyl cellulose (CMC).

### Strain isolation and identification

Pea peels that were purchased locally were used to isolate *Aspergillus awamori*. Two grams of pea peels were soaked for an hour in a 250 Erlenmeyer flask containing 50 ml of sterile distilled water, then, 200 µl was spread on potato dextrose agar plates and plates were incubated at 32 °C for one to five days. To verify the purity of the culture, individual colonies were selected, streaked on potato dextrose agar plates, and then re-incubated. For additional testing, the pure culture was transferred to potato dextrose agar slants. *Aspergillus awamori* 18S rRNA sequence was used for molecular identification by using GeneJET™ PCR Purification Kit (Thermo#K0701) in Sigma Company of Scientific Services, Egypt (www.sigma-co-eg.com).

### Production of cellulase enzyme under solid-state fermentation (SSF)

In the screening step, Seven different agricultural wastes (corn cobs: CC, lemon peels: LP, potato shells: PS, okra suppression: OS, molokhia stem: MS, rice straw: RS, onion peel: OP and pea peels: PP) were used as substrates for cellulase production. Two grams of the dried solid substrates were added to 250 ml Erlenmeyer flasks for solid state fermentation. Moisture was adjusted by addition of 10 ml of distilled water to each flask. Each flask was covered with hydrophobic cotton and autoclaved at 121 °C for 20 min. Following cooling, 1.0 ml of an inoculum containing 5 × 10^7^ spores.ml^− 1^ of a 5-day-old culture was added (measured using spectrophotometer at 625 nm). The inoculum was made by harvesting the fungus’s slant in 20 ml of sterile distilled water. The inoculated flasks were incubated under static conditions for 7 days at 32 ± 2 °C. Every experiment was conducted in duplicate and mean ± SD, which was computed using Microsoft Excel, is the reported average value.

### Extraction of cellulase enzyme

Enzyme extraction was done by adding fifty ml of distilled water and left in a rotary shaker 150 rpm for 60 min. Then the mixture was filtered through a cloth and the culture filtrate centrifuged for 15 min at 5,000 rpm and 4 °C. The supernatant was used as the crude cellulase enzyme extract.

### Enzymes assays

Lignocellulolytic enzymes activities included cellulase, xylanase, pectinase and laccase were determined as mentioned by Mostafa et al. [5] and amylase was determined as mentioned by Ahmed et al. [23] as follows:

#### Cellulase assay

In 0.05 M acetate buffer (pH 5.0), 0.5 ml of 1% carboxymethyl cellulose (CMC) was mixed with half a milliliter of the crude enzyme preparation. After that, the reaction mixture was incubated for 30 min at 50 °C. The Somogyi method [24] was used to determine the released reducing sugars. The amount of enzyme that, under assay conditions, librated one µmol of glucose per minute was defined as one unit of cellulase (IU).

#### Xylanase assay

Half ml of supernatant solution was added to 0.5 ml of 1% xylan in 0.05 M acetate buffer pH 5.0. Then the reaction mixture was incubated at 50 °C for 30 min. The amount of reducing sugar liberated was quantified by the method of Somogyi method [24] using xylose as standard. One unit of xylanase is defined as the amount of enzyme that liberates 1 µmol of xylose equivalents per minute under assay conditions.

#### Pectinase assay

Pectinase activity was evaluated by mixing 0.2 ml enzyme solution and 0.8 ml of 0.5% citrus pectin in 0.05 M acetate buffer (pH 5.0). Samples were incubated for 10 min at 50 °C and the reducing sugars were determined as galacturonic acid by Somogyi method [24]. One unit of enzyme activity was defined as the amount of enzyme releasing1 µmole of galacturonic acid under assay conditions.

#### Amylase assay

Half ml of 1% soluble starch and 0.5 ml of supernatant solution were combined in 0.05 M acetate buffer (pH 5.0) and incubated for 20 min at 40 °C. The Somogyi [24] method was used to determine the released reducing sugars. Under assay conditions, one unit of enzyme activity (IU) was defined as the amount of enzyme that released one µmol of reducing sugar per minute.

#### Laccase assay

The reaction mixture contained 600 µL sodium acetate buffer (0.1 M, pH 5.0 at 27 °C), 300 µL ABTS (5 mM), 300 µL culture filtrate and 1400 µL distilled water. The mixture was then incubated for 2 min. at 30 °C. The absorbance was measured immediately at one-minute intervals at 420 nm. One unit of laccase activity was defined as activity of an enzyme that catalyzes the conversion of 1 µmol of ABTS per minute.

### Optimization of *A. awamori* MK788209 cellulase production by Plackett-Burman design

In this design we investigated the qualitative effect of eleven factors on *Aspergillus awamori* MK788209 cellulase production including A: molokhia stalks (g.flask^− 1^), B: incubation time (days), C: glucose (g.l^− 1^), D: lactose (g.l^− 1^), E: baker’s yeast (g.l^− 1^), F: peptone (g.l^− 1^), G: (NH_4_)_2_SO_4_ (g.l^− 1^), H: CuSO_4_ (g.l^− 1^), J: FeSO_4_(g.l^− 1^), K: CaCl (g.l^− 1^), L: KCl (g.l^− 1^).

Each of these factors was studied with low level (− 1) and high level (+ 1). Total number of experiments was 12 trials based on the rule n + 1, where n: represents the number of factors under investigation (Table 1).

**Table 1:** Plackett-Burman design for *Aspergillus awamori* MK788209 cellulase production

Trial	Factor 1 A:MS weight g/flask	Factor 2 B:inc. time Day	Factor 3 C: glucose g/l	Factor 4 D: lactose g/l	Factor 5 E: baker’s yeast g/l	Factor 6 F: peptone g/l	Factor 7 G: (NH_4_)_2_SO_4_ g/l	Factor 8 H: CuSO_4_ g/l	Factor 9 J: FeSO_4_ g/l	Factor 10 K: CaCl_2_ g/l	Factor 11 L: KCl g/l	Cellulase activity (U/ml)
1	2	4	10	0	1	1	0	0.01	0.01	0.01	0	0.929
2	2	4	0	0	0	0	0	0	0	0	0	1.864
3	2	4	0	10	0	1	1	0	0.01	0.01	0.01	2.053
4	4	7	0	10	1	1	0	0	0	0.01	0	3.727
5	4	7	0	0	0	1	0	0.01	0.01	0	0.01	4.013
6	2	7	0	10	1	0	1	0.01	0.01	0	0	2.522
7	4	4	10	10	1	0	0	0	0.01	0	0.01	2.843
8	4	4	0	0	1	0	1	0.01	0	0.01	0.01	3.507
9	4	4	10	10	0	1	1	0.01	0	0	0	2.613
10	2	7	10	0	1	1	1	0	0	0	0.01	2.369
11	2	7	10	10	0	0	0	0.01	0	0.01	0.01	2.969
12	4	7	10	0	0	0	1	0	0.01	0.01	0	4.636

*Row represents an experiment and column represents an independent variable

The statistical significance was determined by F-value, and the proportion of variance explained by the model obtained was given by the multiple coefficient of determination, R^2^ (Table 2). Experimental responses were analyzed by first order model by the following equation: cellulase activity (U/ml) = β°+ΣβiXi.

**Table 2:** Analysis of variance (ANOVA) for Plackett-Burman design for *Aspergillus awamori* MK788209 cellulase production

Source	Squares	df	Mean Square	F Value	*p*-value Prob > F	
Model	11.20	7	1.600	25.64164	0.0036	Significant
A:MS weight	6.21	1	6.211	99.54612	0.0006	
B:inc. time	3.44	1	3.442	55.17184	0.0018	
E: baker’s yeast	0.42	1	0.422	6.767868	0.0600	
F: peptone	0.58	1	0.579	9.287977	0.0381	
G: (NH_4_)_2_SO_4_	0.15	1	0.153	2.452333	0.1924	
K: CaCl_2_	0.21	1	0.213	3.406518	0.1387	
L: KCl	0.18	1	0.178	2.858838	0.1661	
Residual	0.25	4	0.062			
Cor Total	11.45	11				

Std. Dev. 0.25, R^2^ 0.9782, Mean 2.84, Adj R^2^ 0.9401, C.V. % 8.80, Pred R^2^ 0.8038

,PRESS 2.25, Adeq Precision 17.409

β° is the model intercept and βi is the linear coefficient, and Xi is the level of the independent variable. The experimental design and analysis were done by using (Design Expert) software (version 7.0).

### Optimization of *A. awamori* MK788209 cellulase production by Central Composite Design (CCD)

The most effective parameters from the Plackett-Burman design, namely (A) MS weight (g.flask^− 1^) and (B) incubation time (days), are studied quantitatively in this design. As indicated in Table 3, which was used in CCD, variables were examined at five levels: -1.414, -1, 0, + 1, and + 1.414. This resulted in a total of 13 trials as indicated. Analysis of variance (ANOVA) was used to conduct the model’s statistical analysis (Table 4).

**Table 3:** Central Composite Design for *Aspergillus awamori* MK788209 cellulase production

Trial	Factor 1 A:MS weight (g/flask)		Factor 2 B: incubation time (days)		Cellulase activity (U/ml)	
	Code	Actual	Code	Actual	Predicted	Actual
1	0	6	0	9.5	6.85	6.85
2	-1.414	3.17	0	9.9	5.92	6.15
3	1.414	8.83	0	9.5	8.20	8.12
4	-1	4	1	12	4.61	4.49
5	0	6	0	9.5	6.85	6.85
6	0	6	0	9.5	6.85	6.85
7	0	6	0	9.5	6.85	6.85
8	-1	4	-1	7	8.10	7.85
9	0	6	1.414	13.03	6.69	6.67
10	1	8	1	12	8.90	9
11	1	8	-1	7	7.03	7
12	0	6	-1.414	5.96	7.83	8
13	0	6	0	9.5	6.85	6.85

**Table 4:** Analysis of variance (ANOVA) for CCD design for *Aspergillus awamori* MK788209 cellulase production

Source	Sum of Squares	df	Mean Square	F Value	*p*-value Prob > F	
Model	14.00	5	2.799	113.775	< 0.0001	
A-MS weight	5.19	1	5.189	210.927	< 0.0001	
B-Incubation time	1.30	1	1.303	52.975	0.0002	
AB	7.16	1	7.164	291.192	< 0.0001	Significant
A˄2	0.08	1	0.078	3.161	0.1186	
B˄2	0.29	1	0.295	11.989	0.0105	
Residual	0.17	7	0.025			
Lack of Fit	0.17	3	0.057			
Pure Error	0	4	0			
Cor Total	14.17	12				

Std. Dev. 0.16, R^2^ 0.9878, Mean 7.04, Adj R^2^ 0.9792, C.V. % 2.23,

Pred R^2^ 0.9136, PRESS 1.22, Adeq Precision 40.234

### Applications of *Aspergillus awamori* MK788209 culture filtrate

#### Saccharification of agricultural wastes using *Aspergillus awamori* MK788209 culture filtrate

Eight agricultural wastes were used (CC, LP, PS, OS, MS, RS, OP and PP) In this experiment, each agricultural waste (1 g) was treated with *Aspergillus awamori* MK788209 culture filtrate containing (13 U laccase, 6.67 U cellulase, 23.51 U xylanase, 31.20 U pectinase and 10.91 U amylase) dissolved in 10.00 ml of sodium acetate buffer (0.05 M, pH 5.00) and incubated at 50 °C for 24 h. The amount of reducing sugar released was measured by Somogyi method [24].

#### Improvement of reducing sugar recovery (saccharification) from PP by Central Composite Design (CCD)

The following variables were optimized in this step to maximize the recovery of reducing sugar: A: pea peel weight (g), B: culture filtrate units added (ml), and C: time of saccharification (h) using CCD. Five levels of testing (-1.682, -1.00, 0, + 1.00, and + 1.682) were applied to each factor, yielding fifteen runs (Table 5). Statistical analysis (ANOVA) verified the design’s success (Table 6).

**Table 5:** Central Composite Design for saccharification of PS by *Aspergillus awamori* MK788209 culture filtrate

Run	Factor 1 A: PS weight g	Factor 2 B: enzyme volume ml	Factor 3 C: incubation time h	Reducing sugers mg.ml^− 1^
1	2	10	72	85.15
2	1.375	7.5	89.726	103.66
3	2	5	72	259.109
4	1.375	7.5	46	150.57
5	0.75	5	72	160.89
6	1.375	7.5	46	150.57
7	1.375	7.5	2.273	201.55
8	1.375	7.5	46	150.57
9	1.375	7.5	46	150.57
10	0.323	7.5	46	60.55
11	0.75	10	72	4.336
12	1.375	11.704	46	3.715
13	2	10	20	60.926
14	1.375	3.295	46	281.606
15	1.375	7.5	46	150.57
16	2	5	20	338.183
17	0.75	10	20	45.451
18	2.426	7.5	46	291.636
19	1.375	7.5	46	150.57
20	0.75	5	20	235.614

**Table 6:** Analysis of variance (ANOVA) for CCD design for saccharification of PS by *Aspergillus awamori* MK788209 culture filtrate

Source	Sum of Squares	df	Mean Square	F Value	*p*-value Prob > F	
Model	159590.2168	3	53296.74	79.786	< 0.0001	
A: PS weight	34429.99827	1	34,430	51.542	< 0.0001	
B: enzyme volume	117227.0297	1	117,227	175.490	< 0.0001	significant
C: incubation time	8233.188834	1	8233.189	12.325	0.0029	
Residual	10687.9642	16	667.9978			
Lack of Fit	10687.9642	11	971.6331			
Pure Error	0	5	0			
Cor Total	170578.181	19				

Std. Dev. 25.85, R^2^ 0.9373, Adj R^2^ 0.9256, C.V. % 17.03, Pred R^2^ 0.8840

#### Chromatographic analysis

Hydrolysis product was analyzed by descending paper chromatograph using Whatman No.1 and solvent system n-butanol: acetone: water (4:5:1, v/v/v) [25] and sprayed with aniline phthalate [26].

#### Biopolishing of cotton fabrics

Twill and satin cotton fabrics with and without sodium periodate pretreatment were treated with *A. awamori* MK788209 culture filtrate. Sodium periodate pretreated cotton fabrics were pretreated as followed: One gram of cotton fabric was immersed in 20 ml of 0.05 M sodium periodate solution and reacted at 50 °C. After oxidation, the cotton fabric washed with distilled water for several cycles. Cotton fabrics that had been bleached and oxidized were biopolished using the culture filtrate of *A. awamori* MK788209. This biotreatment was conducted in acetate buffer containing Egyptol (0.5 g.l^-1^) at pH 6.00 and material to liquor ratio 1:50, biotreatment was carried out at concentrations of cellulase enzymes (20 ml.l^-1^), temperatures 50 °C for 45 min in ultrasonic water bath. Then the cotton sample was washed by hot water and dried in an oven at 80 °C. Finally, the fabrics were dried in ambient conditions. Weight loss (%) in fabric weight was calculated from the difference in fabric weight before and after the treatment. Whiteness index was evaluated with a Color-Eye 3100 Spectrophotometer from SDL Inter [27]. Surface roughness was monitored according to JIS 94 standard, using surface roughness measuring instrument, SE 1700a made in Japan. The color strength, expressed as K/S value, of the obtained dyeing’s was measured at the wavelength of maximum absorbance using an automatic filter Spectrophotometer, and calculated by the Kubelka Munk equation [28]: K/S = (1 − R)^2^/2R, where K is the absorption coefficient, S is the scattering coefficient, and R is the reflectance of the dyed samples. Morphology study of the fabrics, the untreated and treated fabrics were analysed by scanning electron microscopy (SEM) and morphological changes of the surface structure.

#### Enzymatic desizing and half bleached treatment

In this experiment one type of cotton fabric was used. With a material to liquor ratio of 1:30, the aqueous desizing solution was made up of enzyme solution (30 ml.l^− 1^), NaClO_2_ (5 g.l^− 1^), and non-ionic wetting agent (1 g.l^− 1^). The reaction was conducted in buffer solution. The fabrics underwent a 30-minute treatment at 60 °C, followed by padding, patching, and another 30 min in oven. They were then twice washed, once with hot water and once with cold water, and dried. The amount of time needed for a water drop to penetrate a piece of fabric was used to estimate wettability. The violet scale shade and TEGEWA scale method were used to evaluate desizing efficiency [29].

#### Coloration of the biotreated cotton samples by dyeing technique

Reactive red dye was used to dye treated cotton fabrics, according to Li et al. [29]. The dyeing process involved the use of sodium sulfate (60 g.l^-1^) for 30 min at 40 °C, followed by a fixation period of 60 min at 60 °C in sodium carbonate (20 g.l^-1^). After dying the samples, they were cleaned and allowed to air dry.

#### Coloration of the biotreated cotton samples by pigment printing technique

Pigment printing was used to print on cotton fabric. Hebeish et al. [30] provided the method for preparing the printing paste. The samples were cleaned in accordance with the guidelines provided in the AATCC test method [31]. The AATCC test procedures were followed to determine the color fastness to rubbing, perspiration, and light [32–34].

### Results and discussion

#### Screening of different agricultural wastes for *A. awamori* MK788209 cellulase production

**Fig. 1:**
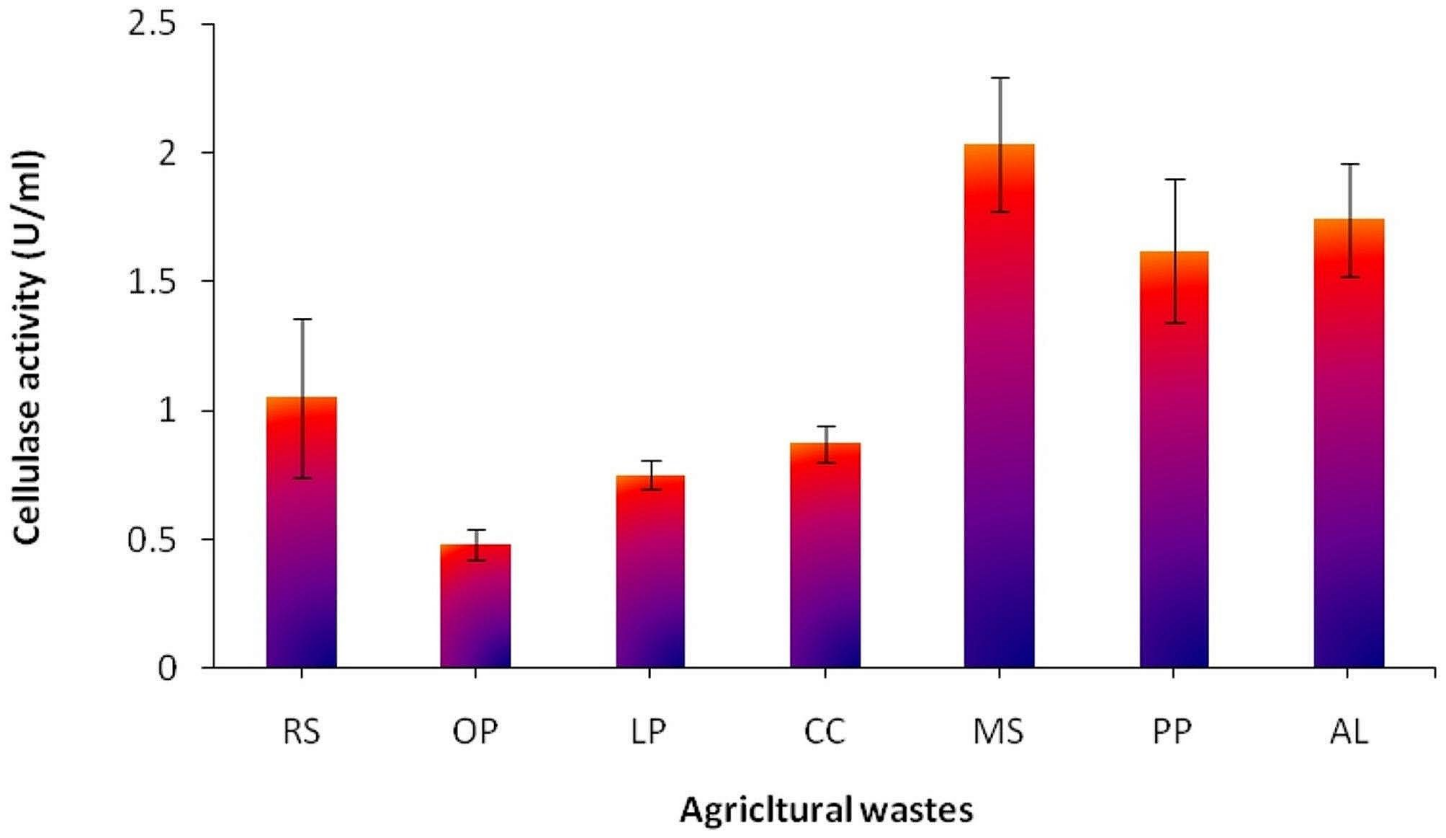
Screening of different agricultural wastes for *A. awamori* MK788209 cellulase production

The nucleotide sequence of the newly isolated endophytic fungus was identified *as A. awamori*, submitted to the GenBank and assigned accession number MK788209. SSF of the aforementioned wastes was used to produce cellulase. Compared to submerged fermentation for fungi, this technique has the advantage of producing higher titres of enzymes [20]. The substrate is a critical key component in any enzyme production process. All of the added agricultural wastes could be utilized by *A. awamori* MK788209, which produced cellulase to varying degrees (Fig. 1). I.e. Cellulase production was in the following order MS > AL > PP > RS > CC > LP > OP. This can be attributed to the different content of lignin of each agricultural waste which is resistant to microbial attack [35] and can impede enzymatic hydrolysis by adhering to cellulose and reducing the amount of enzyme available for enzymatic hydrolysis [36]. Accordingly, MS was found to be the best agricultural waste in our investigation for maximizing cellulase production (2.03 U.ml^− 1^). According to Ahmed et al. [37], MS had a composition of 44% carbohydrates, 22% protein, 16% ash, 5% moisture, 2% fat, 11% fiber, and vitamins. This made MS an ideal solid substrate because it gave the microorganism all the nutrients it needed for maximum production. Many agricultural wastes have been reported to induce the cellulase production as wheat straw [38], wheat bran [39], rice straw [40], Oil palm [41] and sorghum husk [42].

#### Optimization of *Aspergillus awamori* MK788209 cellulase production by Plackett-Burman Design (BPD)

The results, as displayed in Table 1, demonstrated the significant impact of the various factors on cellulase production, with cellulase production varying widely across the twelve trials (0.929–4.636 U.ml^− 1^). Trail 12 produced the highest level of cellulase (4.64 U.ml^− 1^), leading to a 2.51-fold increase. The following formula can be used to calculate the cellulase activity (U.ml^− 1^):

Cellulase activity (U.ml^− 1^) = + 2.84 + 0.72 * molokhia stalks weight + 0.54 * incubation time − 0.19* bakers yeast − 0.22 * peptone + 0.11 * (NH_4_)_2_SO_4_ +0.13 * CaCl + 0.12 * KCl.

**Fig. 2:**
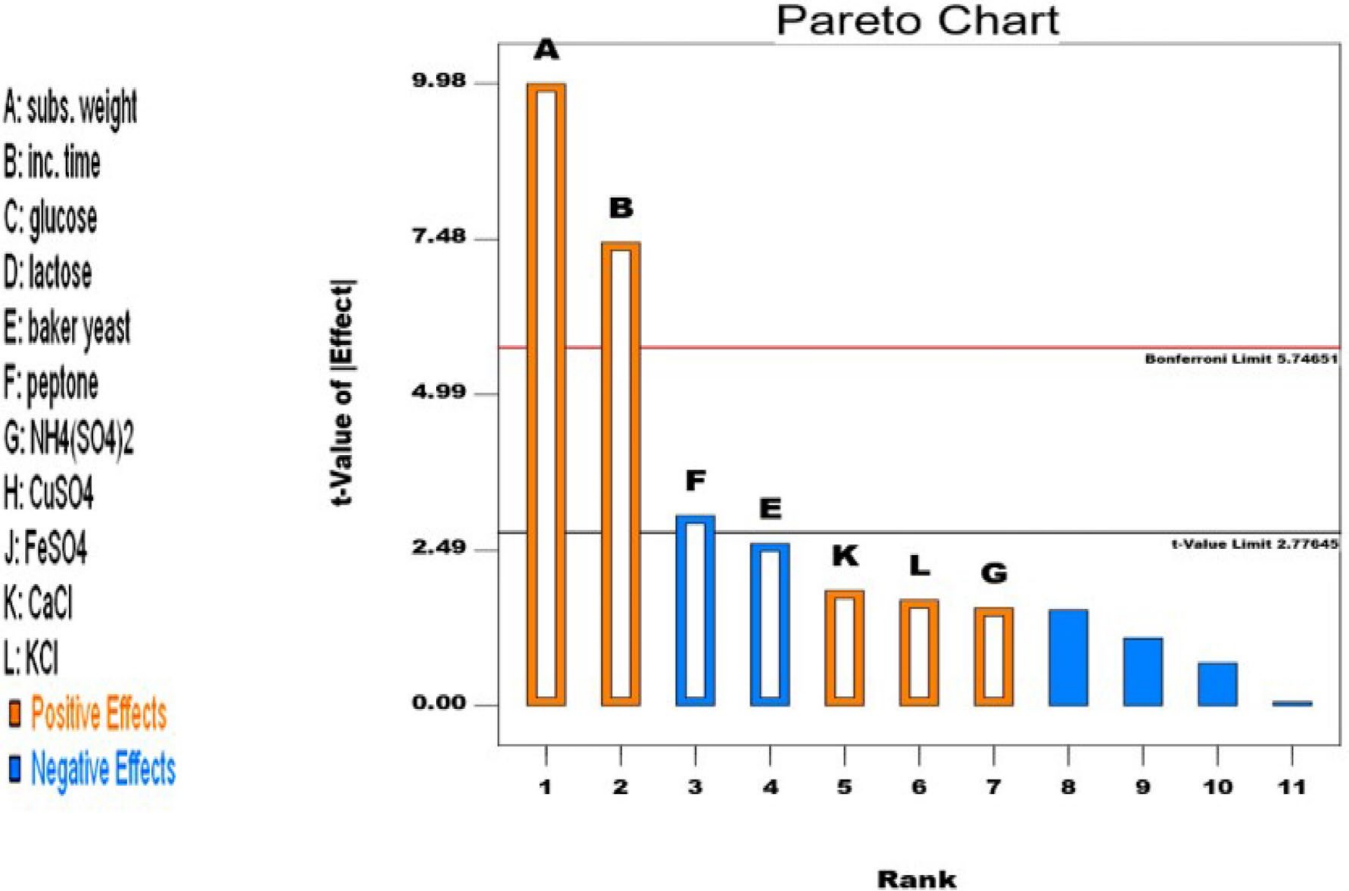
Pareto chart showing the effect of each factor on *A. awamori* MK788209 cellulase production

Only seven of the eleven factors that were examined, including the weight of the molokhia stalks, the incubation period, baker’s yeast, peptone, ammonium sulfate, calcium chloride, and potassium chloride, were found to be significant, as shown in Fig. 2. This result is similar to what Vu et al. [43] reported. The remaining four factors (glucose, lactose, CuSO_4_, and FeSO_4_) had no effect on the production of cellulase by *Aspergillus awamori* MK788209. In contrast to that reported by Sorour et al. [44] and Gunny et al. [45].

The abovementioned seven significant factors were categorized as positive or negative factors affecting *A. awamori* MK788209 cellulase production. The weight of the molokhia stalks, the incubation period, (NH_4_)_2_SO_4_, CaCl, and KCl were the positive factors. On the other hand, peptone and baker’s yeast were negative factors. Molokhia stalk weight and incubation duration had the greatest positive effects on cellulase production out of the five positive significant factors.

The effectiveness of the design for cellulase production was indicated by the analysis of variance as shown in Table 2. The R^2^ value of 0.9782, which shows that 97.82% of the response variability could be explained by the model, validated the design’s variability. The model is successful because the adjusted R^2^ of 0.9401 and the predicted R^2^ of 0.8038 were near to each other. An adequate signal was indicated by the analysis’s adequate precision of 17.409 for cellulase production. Coefficient of variation (CV) was a measure of precision with which experiments were conducted and its value was found to be 8.80. Low CV value predicts accuracy and reliability of the experiments conducted [46].

#### Central Composite Design (CCD)

**Fig. 3:**
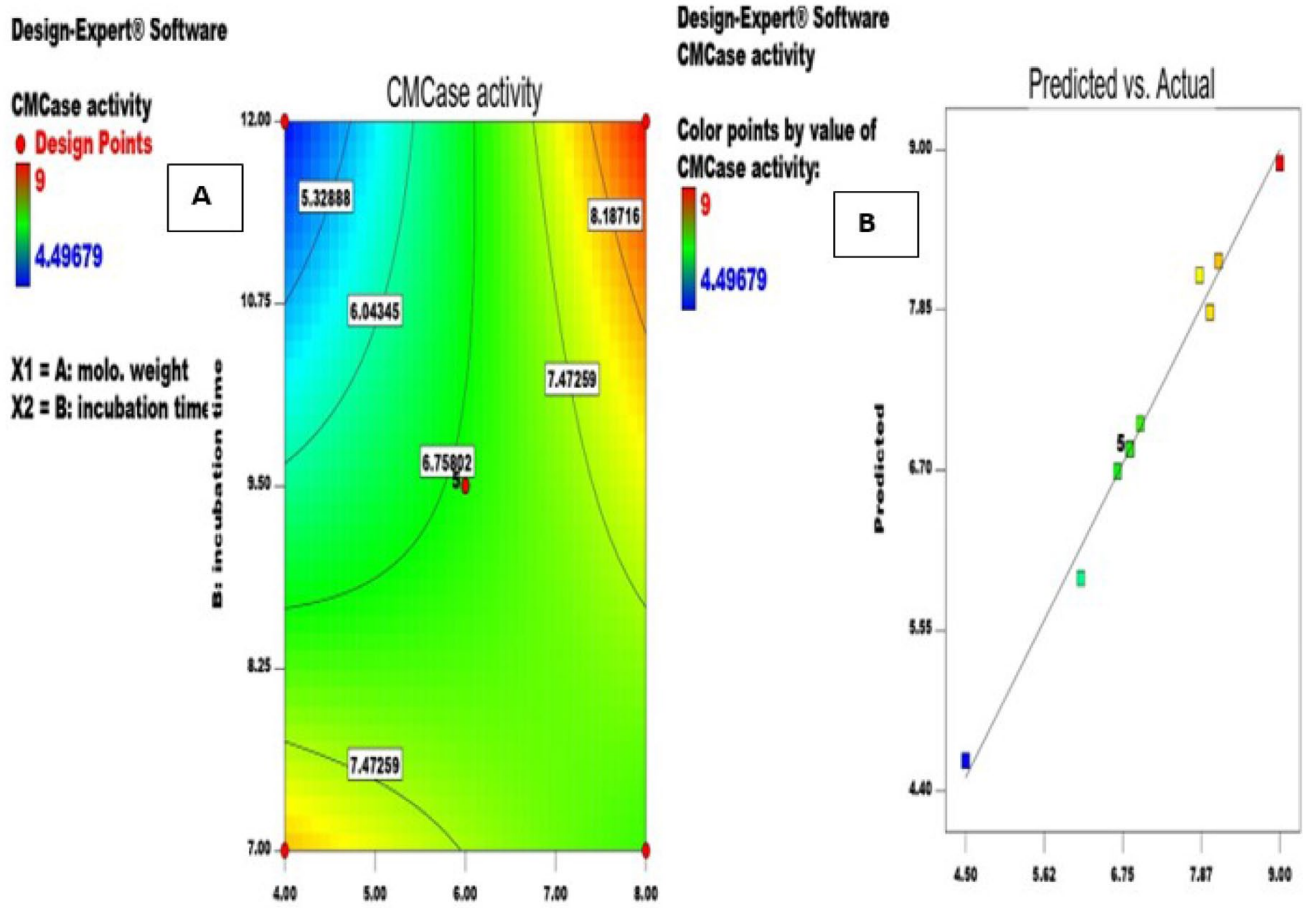
**a**: Contour plot showing interaction between molohkia stalks weight and incubation time; **b**: Parity plot to show the distribution of observed and predicted values for *A. awamori* MK788209 cellulase production

The quantitative impact and interaction between the two most important variables (molohkia stalks weight and incubation time) on *A. awamori* MK788209 cellulase production determined from PB design were shown in Fig. 3a. According to Table 3, trial number 10 produced the maximum cellulase (9.00 U.ml^− 1^). The resulted fold increase was 1.94 and 4.86 fold, when compared to the PBD and unoptimized medium, respectively. Compared to what Premalatha et al. [47] reported, this cellulase yield was higher. Also Verma and Kumar [48] applied Box-Bhenken Design (BBD) to study the levels of three variables (temperature, process pH, inoculum dosages) for cellulase production via SSF of wheat bran by *Aspergillus niger*. In optimization process, the decrease in enzyme production at higher substrate might be due to the microbial growth inhibition. While, enzyme production increases with increase in time period, after a specific time the accumulation of wastes worsens the situation and substrate becomes a limiting factor [38].

The following equation can be used to determine the cellulase activity (U.ml^− 1^):

Cellulase activity (U.ml^− 1^) = + 6.85 + 0.81 * molohkia stalks weight − 0.40 * incubation time + 1.34 * molohkia stalks weight * incubation time + 0.11 * molohkia stalks weight^2^ + 0.21 * incubation time^2^.

Plotting the observed and predicted values of cellulase activity revealed a good agreement between the experimental and predicted values, with the data points localized near the diagonal line in Fig. 3b. The goodness of fit of the model was checked by determination of coefficient R^2^ 0.9878. The closer the R^2^ is to 1.0, the stronger the model and the better it predicted the activity [49]. The adjusted R^2^ value of 0.9792 and the predicted R^2^ value of 0.9136 agreed reasonably well. Table 4 indicates that the model proved to be significant based on its F-value of 113.77. Prob > F values less than 0.0500 suggested that the model terms were significant. Low CV% value 2.23 predicted accuracy and reliability of the experiments conducted [46]. The effectivenes of this design for *Aspergillus awamori* MK788209 cellulase production which indicated by mentioned R^2^, predicated R^2^ and adjusted R^2^ were close to that reported by other authors [45, 47, 50].

#### Saccharification of agricultural wastes by *A. awamori* MK788209 culture filtrate

**Fig. 4:**
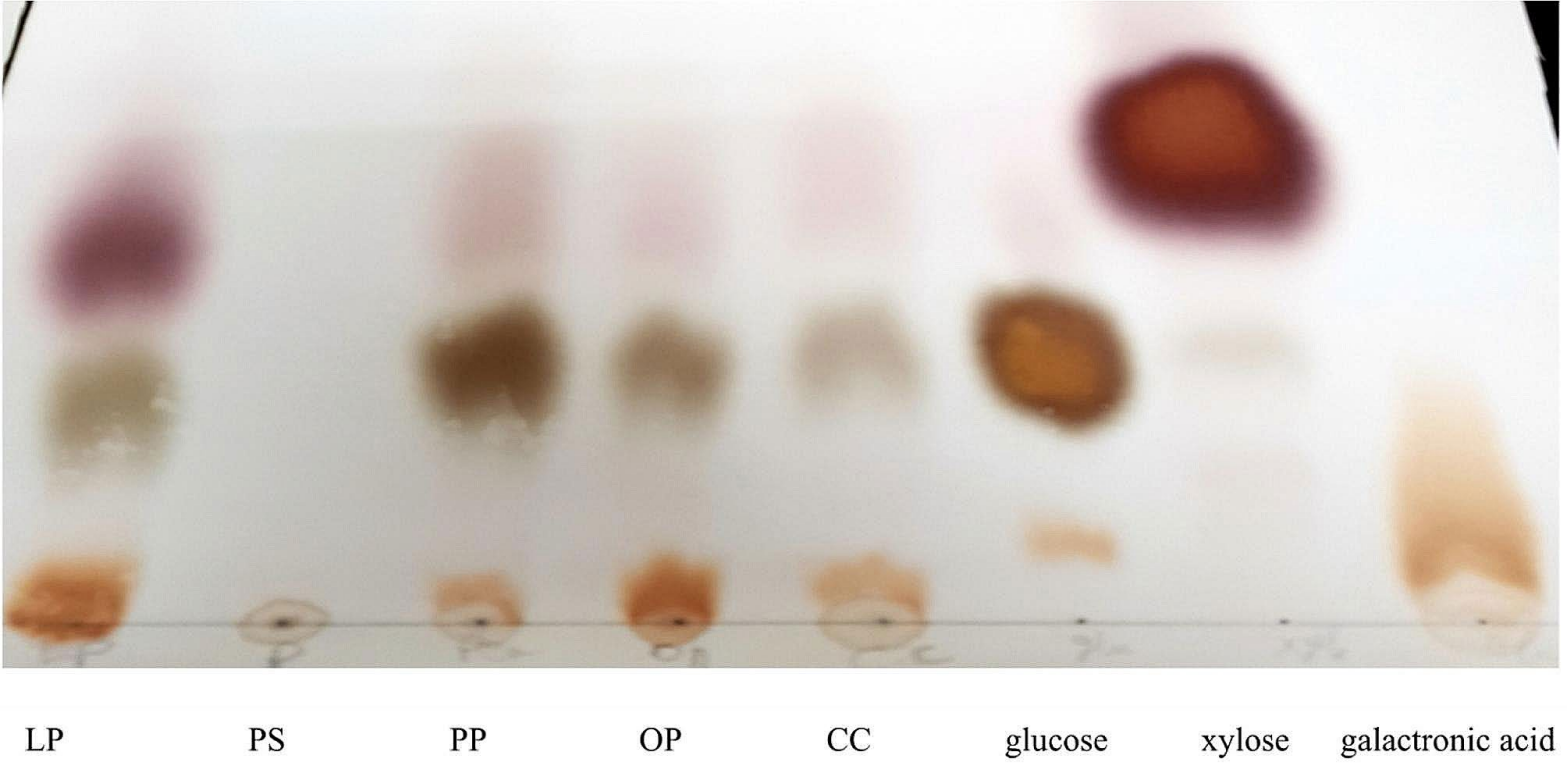
Paper chromatography for enzymatic hydrolysis of different agricultural wastes by *A. awamori* MK788209 culture filtrate

In this experiment, we utilized *A. awamori* MK788209 culture filtrate which was rich in lignocellulolytic enzymes [laccase, cellulase, xylanase, amylase and pectinase] in reducing sugar recovery from agricultural wastes. As shown in (Fig. 4) *A. awamori* MK788209 culture filtrate was capable of hydrolyzing all the agricultural wastes with varied degrees depending on the lignin, cellulose, xylan, starch and pectin contents. The order of sugar recovery was as follow PP > LP > OP > MS > CC > PS > OS > RS. Furthermore, it was demonstrated that a combination of reducing sugars, including glucose, xylose, and galacturonic acid, were produced by the enzymatic hydrolysis of various agricultural wastes. PP produced the highest reducing sugar (98.33 mg.ml^− 1^), which was higher than previously reported [41, 50]. Various lignocellulosic materials were used in several saccharification studies [51–56].

#### Optimization of saccharification by CCD

In general, the RSM optimization outperformed the traditional optimization techniques in improving the experimental results. Using RSM, Tanyildizi et al. [57] and Mahat et al. [58] have reported increases in production yield of 15% and 34%, respectively. Additionally, Santos Gomes [59] used *Penicillium roqueforti* endoglucanase to saccharify sugarcane bagasse, coconut shell, wheat bran, cocoa fruit shell, and cocoa seed husk. They used the Box-Behnken design to assess the effects of three factors on the production of fermentable sugar: substrate concentration, time, and enzyme concentration. They saccharified wheat bran under ideal conditions, (253.19 mg/g of fermentable sugars were obtained), this was 41.5 times more than what was obtained without optimizing. As shown in Table 5 the highest sugar recovery was obtained in trial 16 (338.18 mg.ml^− 1^) causing 3.44-fold increase compared to unoptimized conditions.

The sugar recovery can be calculated from the following equation:

Reducing sugars (mg.ml^− 1^) = + 151.79 + 50.21* A -92.65 * B -24.55* C.

The suceess of the design was emphasized by analysis of (ANOVA) as shown in Table 6. Moreover, The Pred R^2^ of 0.8840 was in reasonable agreement with the Adj R^2^ of 0.9256. The value of R^2^ was 0.9373 meaning that 93.73% of the results can be explained by the design. The model F-value of 79.79 implied the model was significant. There was only a 0.01% chance that a “Model F-Value” this large could occur due to noise.

#### Textile application of *A. awamori* MK788209 culture filtrate

The results of treating textile samples with *A. awamori* MK788209 culture filtrate varied depending on two main factors, as indicated in Table 7: the type of fabric sample and fabric pretreatment with sodium periodate. Compared to satin, the treatment of twill fabric with *A. awamori* MK788209 culture filtrate had a greater positive impact. The surface became smoother in both instances, as seen in Fig. 4b and d. This could be because cellulase hydrolyzed easily accessible pills or fibrils at the surface of the treated substrate. These findings were superior to those published by Abd El Aty et al. [6] and consistent with other earlier research [60]. The cotton fibers’ surface morphology appeared to be completely unaltered after being treated with the *A. awamori* MK788209 culture filtrate, according to scanning electron micrographs of the samples.

**Table 7:** Effect of enzymatic treatments on some physicochemical an color properties of cotton fabrics

Cotton Fabric type		WL%	Roughness (µm)	W.I.	K/S
Twill	Untreated (bleached)	2.19	11.8	78.37	12.22
	Treated (oxidized)	2.74	13.92	71.37	14.18
	Blank	-	24.7	71.73	8.63
Satin	Untreated (bleached)	2.14	10.8	64.34	12.08
	Treated (oxidized)	2.59	12.59	65.26	21.85
	Blank	-	11.75	68.80	11.73

^Untreated: Enzymatic treated only^

^Treated: cotton fabric sample pretreated with sodium periodate followed by enzymatic treatment^

Additionally, the results in Table 7 demonstrated how *A. awamori* MK788209 culture filtrate affected some color characteristics of twill and satin fabric samples. These color properties, expressed as K/S values, showed that enzymatic treatment of the twill and satin fabric improved their printing ability. The bio-treated fabric’s higher K/S can likely be attributed to the formation of new dye-absorbing surfaces, changes in pore structure, and simultaneous removal of fibrillar matter, which increase the amount of dye diffusion and penetration into the treated fabric’s structure and fixation of dye molecules.

Also, periodate pretreatment followed by enzymatic treatment improved the dyeability of twill and satin fabrics compared to the bleached twill and satin fabrics. This can be explained by the fact that sodium periodate produced several aldehyde groups in cotton and reduced its crystallinity. The aldehyde groups enhanced cellulase binding to cotton substrates, which in turn enhanced dyability [61, 62]. These outcomes were almost identical to those reported by Abd El Aty et al. [6] for the textile application of *Trichoderma longibrachiatum* KT693225 cellulase on cotton (white), linen (white), and indigo-dyed fabrics. Furthermore, after treating twill fabric with *A. awamori* MK788209 culture filtrate, a significant improvement in the whiteness index was noted.

**Fig. 5:**
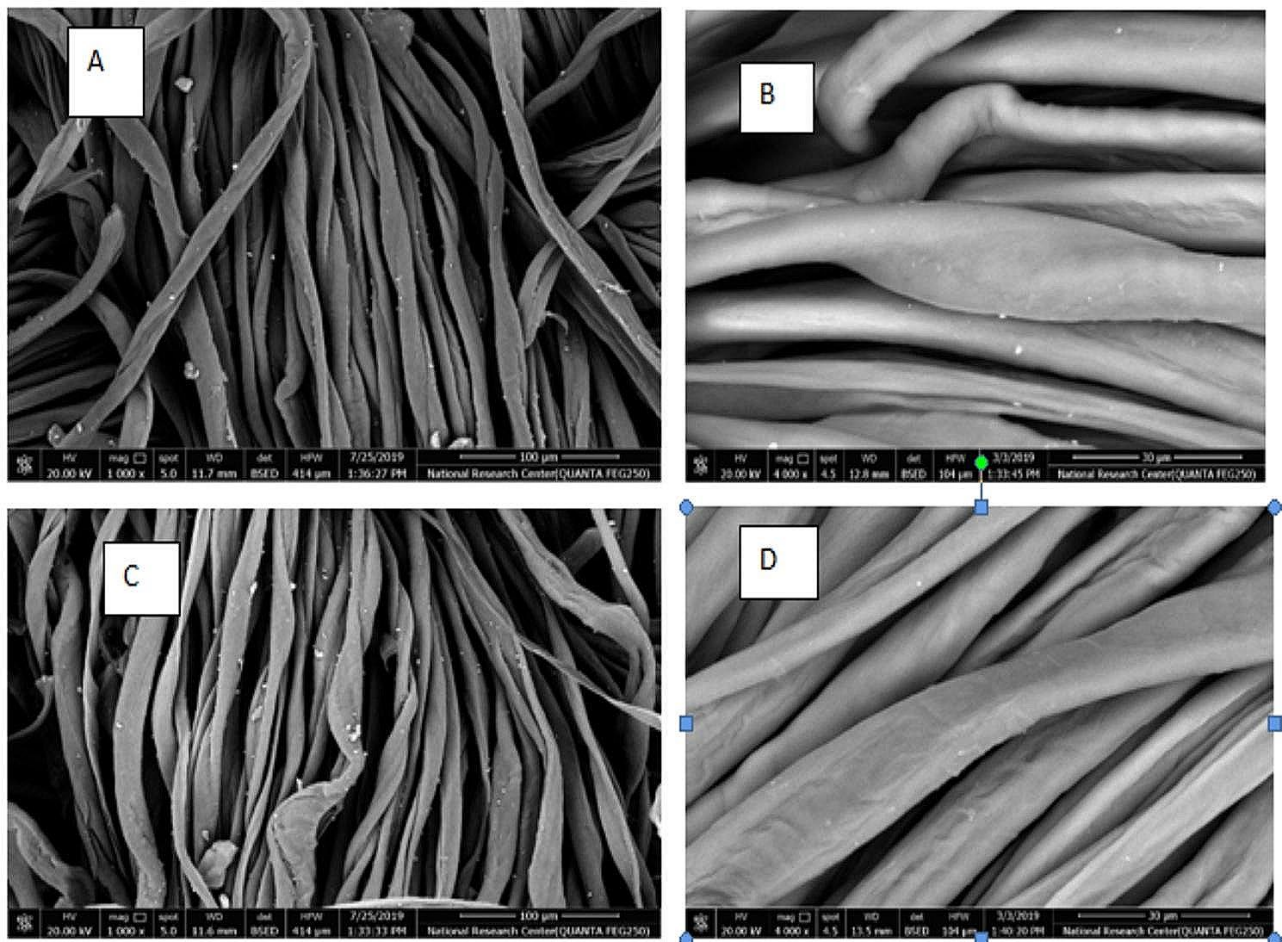
SEM for **a**: untreated twill cotton fabric **b**: *A. awamori* culture filtrate treated twill cotton fabric **c**: untreated satin cotton fabric **d**: *A. awamori* culture filtrate treated satin cotton fabric

Moreover, the desizing of cotton fabric showed 5.2% weight loss, 61 whitness index and violet scale shade of 7. These results were similar to that declared by Abd El Aty et al. [6]. Scanning electron micrographs of the samples (Fig. 5) showed the starch particles on the surface of the fibers of the sized sample, whereas the sample treated with *A. awamori* MK788209 culture filtrate had no starch particle adhered to its surface. This finding confirmed the ability of *A. awamori* MK788209 culture filtrate to desize cotton fabrics. Yahya et al. [63] reported 9% weight loss and TEGEWA scale 9 of gray cotton desizing by amylase from *A. tubingensis* SY 1.

The dyed fabric treated with enzyme showed K/S of 2.87. This high K/S value may be due to enzyme action on fabric impurities leading to cleaner surface and modification of the pore microstructure. Thus, by thermo-fixation the binder film formed covered the entire fiber surface [64].

Curcumine dye was used in cotton printing as an eco- friendly dye with well known antimicrobial activity. The biotreated printed fabric had K/S value of 1.25. The fastness properties presented in Table 8 showed that washing and perspiration fastness properties of fabrics ranged from good to very good. While, light fastness properties ranged from very good to excellent.

**Table 8:** Fastness properties of biotreated dyed and printed cotton samples

Coloration method	Color fastness				Perspiration fastness				Light fastness
	Rubbing		Washing		Acid		Alkaline		
	Wet	Dry	Alt	St	Alt	St	Alt	St	
Dyed 1	3–4	3–4	3–4	3–4	3–4	3–4	2–3	2–3	5
Dyed 2	4–5	4–5	4–5	4–5	4	4	3–4	3–4	5
Printed 1	3	2–3	3–4	3–4	3–4	3–4	2–3	2–3	4–5
Printed 2	4–5	4–5	3	3–4	3	4	3–4	3–4	5

^Dyed 1 and Printed 1: cotton samples biotreated with *A. awamori* MK788209 culture filtrate^

^Dyed 2 and Printed 2: cotton samples biotreated with commercial enzyme^

Cellulase was well-produced by *Aspergillus awamori* MK788209 utilizing a variety of lignocellulolytic wastes, particularly molokhia stems. As *A. awamori* culture filtrate was rich in lignocellulolytic enzymes, the saccharification process of agricultural wastes yielded reducing sugar which can be very beneficial in bioethanol industry. Moreover, *A. awamori* culture filtrate showed high effectiveness in improving the physicochemical and color properties in treatment of twill and satin cotton fabrics.

## Data Availability

Not applicable.

## Abbreviations

*A. awamori*: *Aspergillus awamori*
SSF: Solid state fermentation
PP: Pea peels
CCD: Central Composite Design
PB: Plackett-Burman design
CC: Corn cobs
LP: Lemon peels
PS: Potato shells
OS: Okra suppression
MS: Molokhia stem
RS: Rice straw
OP: Onion peel
CV: Coefficient of variation

## References

[1] Polizeli MD, Rizzatti AC, Monti R, Terenzi HF, Jorge JA, Amorim DD (2005). Xylanases from fungi: properties and industrial applications. Appl Microbiol Biotechnol.

[2] Kang SW, Park YS, Lee JS, Hong SI, Kim SW (2004). Production of cellulases and hemicellulases by *Aspergillus Niger* KK2 from lignocellulosic biomass. Bioresour Technol.

[3] Himmel ME, Xu Q, Luo Y, Ding SY, Lamed R, Bayer EA (2010). Microbial enzyme systems for biomass conversion: emerging paradigms. Biofuels.

[4] Liu P, Lin A, Zhang G, Zhang J, Chen Y, Shen T, Zhao J, Wei D, Wang W (2019). Enhancement of cellulase production in *Trichoderma reesei* RUT-C30 by comparative genomic screening. Microb Cell Fact.

[5] Mostafa FA, Abd El Aty AA, Hamed ER, Eid BM, Ibrahim NA (2016). Enzymatic, kinetic and anti-microbial studies on aspergillus terreus culture filtrate and Allium cepa seeds extract and their potent applications. Biocatal Agric Biotechnol.

[6] Abd El Aty AA, Saleh SA, Eid BM, Ibrahim NA, Mostafa FA (2018). Thermodynamics characterization and potential textile applications of Trichoderma longibrachiatum KT693225 xylanase. Biocataly Agricultur Biotechnol.

[7] Toushik SH, Lee KT, Lee JS, Kim KS (2017). Functional applications of lignocellulolytic enzymes in the fruit and vegetable processing industries. J Food Sci.

[8] Pothiraj C, Kanmani P, Balaji P (2006). Bioconversion of lignocelluloses materials. Mycobiology.

[9] Singh DP, Prabha R, Renu S, Sahu PK, Singh V (2019). Agrowaste bioconversion and microbial fortification have prospects for soil health, crop productivity, and ecoenterprising. Int J Recycl Organic Waste Agric.

[10] Bertero M, de la Puente G, Sedran U (2012). Fuels from bio-oils: bio-oil production from different residual sources, characterization and thermal conditioning. Fuel.

[11] Sànchez C (2009). Lignocellulosic residues: biodegradation and bioconversion by fungi. Biotechnol Adv.

[12] Huang DL, Zeng GM, Feng CL, Hu S, Lai C, Zhao MH, Su FF, Tang L, Liu HL (2010). Changes of microbial population structure related to lignin degradation during lignocellulosic waste composting. Bioresour Technol.

[13] Singh S, Nain L (2014). Microorganisms in the conversion of agricultural wastes to compost. Proc Ind Natl Sci Acad.

[14] Singh DP, Prabha R. Bioconversion of agricultural wastes into high value biocompost: a route to livelihood generation for farmers. Adv Recycl Waste Manag 20172 (3), 10.4172/2475-7675.100013.

[15] Sudharmaidevi CR, Thampatti KC, Saifudeen N (2017). Rapid production of organic fertilizer from degradable waste by thermochemical processing. Int J Recycl Org Waste Agric.

[16] Milala MA, Shugaba A, Gidado A, Ene AC, Wafar JA (2005). Studies on the use of agricultural wastes for cellulase enzyme productions by *Aspergillus Niger*. Res J Agric Biol Sci.

[17] Bansal N, Tewari R, Soni R, Soni (2011). SK A novel strain of *Aspergillus Niger* producing a cocktail of industrial depolymerising enzymes for the production of second generation biofuels. BioRes.

[18] Deswal D, Khasa YP, Kuhad RC (2011). Optimization of cellulase production by a brown rot fungus *Fomitopsis Sp* RCK2010 under solid state fermentation. Bioresour Technol.

[19] Ahmed SA, El-Shayeb N, Hashem AG, Saleh SA, Abdel-Fattah AF (2015). Chemical modification of *Aspergillus Niger* β-glucosidase and its catalytic properties. Braz J Microbiol.

[20] Behera SS, Ray RC (2016). Solid state fermentation for production of microbial cellulases: recent advances and improvement strategies. Int J Biol Macromol.

[21] Duff SJ, Murray WD (1996). Bioconversion of forest products industry waste cellulosics to fuel ethanol: a review. Bioresour Technol.

[22] Kumar D, Bhardwaj R, Jassal S, Goyal T, Khullar A, Gupta N (2021). Application of enzymes for an eco-friendly approach to textile processing. Environ Sci Pollut Res.

[23] Ahmed SA, Mostafa FA, Helmy WA, Abdel-Naby MA (2017). Improvement of bacterial α-amylase production and application using two steps statistical factorial design. Biocataly Agricultur Biotechnol.

[24] Somogyi MJ (1952). Notes on sugars determinations. J Biol Chem.

[25] Tanaka T, Oi S, Iizuka M, Yamamoto T, Levansucrase of. *Bacillus subtilis*. Agric. Biol.Chem. 1978;42(2):323–326 10.1271/bbb1961.42.323.

[26] Block RJ, Durrum EL, Zweig UA. Manual of paper chromatography and paper electrophoresis, Academic Press, New York, 1955;127. 10.1002/ange.19550671421.

[27] Welch CM, Peters JG (1997). Mixed polycarboxylic acids and mixed catalyst in formaldehyde-free durable press finishing. Text Chem Colorist.

[28] Giles CH, Sinclair R, Duff DG (1989). Giles’s laboratory course in dyeing.

[29] Li S, Boyter H, Stewart N. Ultraviolet (UV) curing for textile coloration. AATCC Rev. 2004(8):44–9.

[30] Hebeish A, Ragheb AA, Rekaby M, El-Hennawi HM, Shahin AA (2019). Chitosan/Disperse dye nanoparticles for concomitant printing and antibacterial finishing. Nanotechnol Russ.

[31] Test Method AATCC. (36-1972), 68. Colorfastness to Washing: Characterizationof Textile Colorants, Technical Manual Method of the American Association of TextileChemists and Colorists, USA. (1993).

[32] Test Method. (8-1989), 68. Colorfastness to Crocking: AATCC Crockmeter Method, Technical Manual Method of the American Association of Textile Chemists and Colorists, USA. (1993).

[33] Test Method AATCC. (15-1989), 68. Colorfastness to Perspiration, TechnicalManual Method of the American Association of Textile Chemists and Colorists, USA. (1993).

[34] Test Method. (16A-1989), 68. Colorfastness to Light: Outdoor, TechnicalManual Method of the American Association of Textile Chemists and Colorists, USA. (1993).

[35] Loow Y-L, New EK, Yang GH, Ang LY, Foo LYW, Wu TY (2017). Potential use of deep eutectic solvents to facilitate lignocellulosic biomass utilization and conversion. Cellulose.

[36] Zhu L, O’Dwyer JP, Chang VS, Granda CB. Structure features affecting biomass enzymatic digestibility. Bioresour Technol 2008;99:3817–28. 10.1016/j.biortech.2007.07.033. 10.1016/j.biortech.2007.07.033 17826088

[37] Ahmed SA, Saleh SA, Mostafa FA, Abd El Aty AA, Ammar HA (2016). Characterization and valuable applications of xylanase from endophytic fungus *aspergillus terreus* KP900973 isolated from *Corchorus olitorius* Biocataly. Agricultur Biotechnol.

[38] Shahid ZH, Irfan M, Nadeem M, Syed Q, Qazi JI (2016). Production, purification, and characterization of carboxymethyl cellulase from novel strain *Bacillus megaterium*. Environ Prog Sustain Energy.

[39] Bansal N, Tewari R, Gupta JK, Soni R, Soni SK (2012). Production of cellulases from *aspergillus Niger* NS-2 in solid state fermentation on agricultural and kitchen waste residues. Waste Manag.

[40] Narra M, Dixit G, Divecha J, Madamwar D, Shah AR. Production of cellulases by solid state fermentation with *aspergillus terreus* and enzymatic hydrolysis of mild alkali-treated rice straw. Bioresource Technol 2012;121:355–61. 10.1016/j.biortech.2012.05.140. 10.1016/j.biortech.2012.05.140 22864171

[41] Ang SK, Shaza EM, Adibah Y, Suraini AA, Madihah MS. Production of cellulases and xylanase by *aspergillus fumigatus* SK1 using untreated oil palm trunk through solid state fermentation. Process Biochem 2013;48:1293–302. 10.1016/j.procbio.2013.06.019.

[42] Waghmare PR, Patil SM, Jadhav SL, Jeon BH, Govindwar SP. Utilization of agricultural waste biomass by cellulolytic isolate *Enterobacter sp* SUK-Bio. Agricul Nat Resourc 2018;52:399–406 10.1016/j.anres.2018.10.019.

[43] Vu VH, Pham TA, Kim K (2011). Improvement of fungal cellulase production by mutation and optimization of solid state fermentation. Mycobiology.

[44] Sorour AA, Olama ZA, El-Naggar MY, Ali SM (2023). Bioprocess development for extraction and purification of cellulases from *Aspergillus Niger* 3ASZ using statistical experimental design techniques International. J Biol Macromol.

[45] Gunny AA, Arbain D, Jamal P, Gumba RE (2015). Improvement of halophilic cellulase production from locally isolated fungal strain. Saudi J Biol Sci.

[46] Sen R, Swaminathan T (2004). Response surface modeling and optimization to elucidate and analyze the effects of inoculums age and size on surfactin production. Biochem Eng J.

[47] Premalatha N, Gopal NO, Jose PA, Anandham R, Kwon SW. Optimization of cellulase production by *Enhydrobacter* sp. ACCA2 and its application in biomass saccharification. Frontiers in Microbiology. 2015;6:1046. 10.3389/fmicb.2015.01046. 10.3389/fmicb.2015.01046 PMC4597110 26500615

[48] Verma N, Kumar V (2019). Application of Box–Behnken design for the optimization of cellulase production under solid-state fermentation. SN Appl Sci.

[49] Chen H, Wu MB, Chen ZJ, Wang ML, Lin JP, Yang LR (2013). Enhancing production of a 24-membered ring macrolide compound by a marine bacterium using response surface methodology. J Zhejiang Univ Sci B.

[50] Annamalai N, Rajeswari MV, Balasubramanian T (2014). Enzymatic saccharification of pretreated rice straw by cellulase produced from *Bacillus carboniphilus* CAS 3 utilizing lignocellulosic wastes through statistical optimization. Biomass Bioenergy.

[51] Maleki M, Shahraki MF, Kavousi K, Ariaeenejad S. Salekdeh GH A novel thermostable cellulase cocktail enhances lignocellulosic bioconversion and biorefining in a broad range of pH. Int J Biol Macromol 2020;154:349–60 10.1016/j.ijbiomac.2020.03.100. 10.1016/j.ijbiomac.2020.03.100 32179121

[52] Zhang Q, Cai W. Enzymatic hydrolysis of alkali-pretreated rice straw by *Trichoderma reesei* ZM4-F3. Biomass Bioenergy 2008;32:1130–5. 10.1016/j.biombioe.2008.02.006.

[53] Ma H, Liu WW, Chen X, Wu YJ, Yu ZL (2009). Enhanced enzymatic saccharification of rice straw by microwave pretreatment. Bioresour Technol.

[54] Bak JS, Ko JK, Han YH, Lee BC, Choi IG, Kim KH (2009). Improved enzymatic hydrolysis yield of rice straw using electron beam irradiation pretreatment. Bioresour Technol.

[55] Sukumaran RK, Singhania RR, Mathew GM. Pandey a cellulase production using biomass feed stock and its application in lignocelluloses saccharification for bio-ethanol production. Renew Energy 2009;34:421–4. 10.1016/j.renene.2008.05.008.

[56] Zhang H, Wu J (2022). Statistical optimization of aqueous ammonia pretreatment and enzymatic saccharification of corn stover for enhancing sugars production. Environ Technol Innov.

[57] Tanyıldızı MS, Elibol M, Ozer D (2006). Optimization of growth medium for the production of α-amylase from *Bacillus amyloliquefaciens* using response surface methodology. J Chem Technol Biotechnol.

[58] Mahat MK, Illias RM, Rahman RA, Abd Rashid NA, Mahmood NA, Hassan O, Aziz SA, Kamaruddin K (2004). Production of cyclodextrin glucanotransferase (CGTase) from alkalophilic *Bacillus sp* TS1-1: media optimization using experimental design. Enzyme Microb Technol.

[59] Santos Gomes MM, Nicodemos IS, Costa Silva MD, Santos DM, Santos Costa F, Franco M, Pereira HJ. Optimization of enzymatic saccharification of industrial wastes using a thermostable and halotolerant endoglucanase through Box-Behnken experimental design. Preparative Biochem Biotechnol 2023 Apr 15:1–1. 10.1080/10826068.2023.2201936. 10.1080/10826068.2023.2201936 37071540

[60] Ibrahim NA, El-Badry K, Eid BM. Hassan TM a new approach for biofinishing of cellulose-containing fabrics using acid cellulases. Carbohydr Polym 2011;83:116–21. 10.1016/j.carbpol.2010.07.025.

[61] Ramadan MA, Samy S, Abdulhady M, Hebeish AA (2011). Eco-friendly pretreatment of cellulosic fabrics with chitosan and its influence on dyeing efficiency. Nat Dyes Intech Open.

[62] Hao L, Wang R, Zhao Y, Fang K, Cai Y. The enzymatic actions of cellulase on periodate oxidized cotton fabrics. Cellulose 2018;25:6759–69. 10.1007/s10570-018-2016-8.

[63] Yahya S, Sohail M, Khan SA (2022). Characterization, thermal stabilization and desizing potential of amylase from A. tubingensis SY 1. J Text Inst.

[64] Kumar M, Shukla SR, Arputharaj A, Saxena S, Patil S, Patil PG, Varghese E, Amarowicz R. Biopolishing of cellulosic fabrics: a study on low-stress mechanical properties, microstructure, and dye uptake. 2021 Fibers Polym 22:2803–14. 10.1007/s12221-021-0356-8.

